# B Cells Influence Encephalitogenic T Cell Frequency to Myelin Oligodendrocyte Glycoprotein (MOG)_38–49_ during Full-length MOG Protein–Induced Demyelinating Disease

**DOI:** 10.4049/immunohorizons.2400069

**Published:** 2024-09-27

**Authors:** Michael A. Faust, Lisa Gibbs, Juan M. Oviedo, Douglas H. Cornwall, Keke C. Fairfax, Zemin Zhou, Tracey J. Lamb, Brian D. Evavold

**Affiliations:** Division of Microbiology and Immunology, Department of Pathology, University of Utah, Salt Lake City, UT

## Abstract

Although T cells are encephalitogenic during demyelinating disease, B cell–depleting therapies are a successful treatment for patients with multiple sclerosis. Murine models of demyelinating disease utilizing myelin epitopes, such as myelin oligodendrocyte glycoprotein (MOG)_35–55_, induce a robust CD4 T cell response but mitigate the contribution of pathological B cells. This limits their efficacy for investigating how B cell depletion affects T cells. Furthermore, induction of experimental autoimmune encephalomyelitis with a single CD4 T cell epitope does not reflect the breadth of epitopes observed in the clinic. To better model the adaptive immune response, mice were immunized with the full-length MOG protein or the MOG_1–125_ extracellular domain (ECD) and compared with MOG_35–55_. Mature MOG-reactive B cells were generated only by full-length MOG or ECD. The CNS-localized T cell response induced by full-length MOG is characterized by a reduction in frequency and the percentage of low-affinity T cells with reactivity toward the core epitope of MOG_35–55_. B cell depletion with anti-CD20 before full-length MOG-induced, but not ECD-induced, demyelinating disease restored T cell reactivity toward the immunodominant epitope of MOG_35–55_, suggesting the B cell–mediated control of encephalitogenic epitopes. Ultimately, this study reveals that anti-CD20 treatment can influence T cell epitopes found in the CNS during demyelinating disease.

## Introduction

Multiple sclerosis (MS) occurs with immune-mediated myelin sheath degradation. MHC class II alleles are major risk factors for MS and underlie a critical pathogenic role for CD4 T cells (1–4). During MS, proinflammatory CD4 T cells respond to epitopes derived from myelin-associated proteins (5–9). The ability of CD4 T cells to transfer demyelinating disease in murine experimental autoimmune encephalomyelitis (EAE) confirms that T cell recognition of myelin epitopes is sufficient to initiate paralysis (10–12). Despite the proinflammatory role of CD4 T cells in MS, B cell depletion has been a remarkably successful treatment strategy (13–17). Although Ab production is a unique function of class-switched B cells, the improvement of patient well-being with unchanged IgG levels suggests an Ab-independent mechanism (13, 18). Evidence of B cell Ag presentation of myelin-derived epitopes has been observed in humans (5, 6, 19–21) and mice (21–25), indicating a pathological link between B cells and CD4 T cells. To date, how B cells influence the myelin-reactive CD4 T cell repertoire is not fully understood.

Mouse models of EAE often consist of immunization with myelin-derived peptide fragments that break tolerance by activating CNS-homing pathogenic CD4 T cells and bypassing the typical Ag processing fate of MOG by APCs (26). One of the most well-described models includes the immunization of C57BL/6J (B6) mice (IA^b^-restricted MHC class II) with myelin oligodendrocyte glycoprotein (MOG) peptide fragment 35–55 (MOG_35–55_). Its core IA^b^-restricted T cell epitope is MOG_38–49_ (27–29), and CD4 T cells are typically identified using IA^b^:MOG_38–49_ tetramers (28–31). However, the bulk of autoimmune CD4 T cells express TCRs that are of too low affinity for detection by tetramers and require detection by other means. The two-dimensional micropipette adhesion frequency assay (MP) is the most sensitive method for quantification of low-affinity CD4 T cells (28, 30, 32–38). Previous work identified that most CD4 T cells in the CNS following MOG_35–55_ priming are reactive to IA^b^:MOG_38–49_ in B6 or IA^g7^:MOG_42–55_ in NOD mice (30, 32–34).

Using any myelin-associated peptide induction strategy may underestimate the significance of epitope diversity during demyelinating disease. MOG is a 218-aa transmembrane protein with multiple known IA^b^-restricted epitopes (27, 39, 40). Most notably, the MOG transmembrane domain contains epitopes exhibiting encephalitogenic properties in mice and humans (39–41). To increase epitope diversity, mice were challenged with full-length MOG protein (full-length MOG) or the MOG_1–125_ extracellular domain (ECD) and the effect on the ensuing adaptive immune response was studied. Priming with either full-length MOG or ECD each generated a mature, MOG-reactive B cell response. However, B cell ablation with anti-CD20 before EAE induction delayed the onset of paralysis only after full-length MOG immunization. Multiepitope full-length MOG-induced EAE resulted in a decrease in T cells reactive toward MOG_38–49_ relative to single-epitope MOG_35–55_ and ECD, and anti-CD20 treatment restored the frequency of MOG_38–49_-reactive T cells during full-length MOG-induced EAE. Taken together, the data suggest that B cells expand the range of encephalitogenic CD4 T cells. B cell–targeting therapies for demyelinating disease may therefore reduce the repertoire of pathogenic CD4 T cells by decreasing T cell epitope diversity.

## Materials and Methods

### Mice

Female B6 mice (8–12 wk of age) were purchased from The Jackson Laboratory (Bar Harbor, ME) and housed in specific pathogen-free conditions in accordance with Institutional Animal Use and Care Committee at the University of Utah.

### Recombinant protein production

C-terminal FLAG-tagged constructs encoding mouse full-length MOG protein (MOG) or its ECD were each cloned into a pWPI lentiviral vector (Addgene). High-titer lentiviral supernatants were generated by transfection of a 293T packaging cell line (American Type Culture Collection) with pWPI-MOG or pWPI-ECD, as well as the packaging plasmids pMD2.G and psPAX2 (Addgene). MOG or ECD lentiviral supernatant-transduced 293T cells, a human embryonic kidney cell line lacking endogenous MOG expression, were sorted for high expression of GFP using a FACSAria cell sorter (BD Biosciences). GFP indicated stable expression of MOG or ECD. Each was purified using an anti-FLAG M2 affinity column (MilliporeSigma). The protein eluate was supplemented with n-octyl-β-d-glucopyranoside (MilliporeSigma) to aid in the solubility of the transmembrane region. Eluates were subjected to buffer exchange into sterile 1× PBS (pH 7). Protein purification was confirmed by Coomassie Blue stain and Western blots using anti-FLAG Ab (Sigma-Aldrich) before immunizations.

### MOG immunizations

For EAE inductions, 200 µg of MOG_35–55_ (ABI Scientific), 100 µg of MOG, or 100 µg of ECD were emulsified via sonication into IFA (BD Biosciences) supplemented with 5 mg/ml *Mycobacterium tuberculosis* (BD Biosciences). Mice received s.c. injections of emulsion at day 0 in addition to i.p. injections of 300 ng of pertussis toxin (List Biological) at days 0 and 2. Clinical scores and weights were monitored every 2 d until the onset of symptoms, at which point daily monitoring was implemented. For B cell depletion studies, mice were treated with i.p. injections of 100 µg of anti-CD20 (clone MB20-11) or anti-dengue IgG2c isotype control (Bio X Cell) weekly at days −15, −8, and −1 prior to immunizations. Mice were scored according to the following scale: 1, limp tail; 1.5, single leg inability to grasp cage grate; 2, dual leg inability to grasp cage grate; 2.5, complete paralysis in single hindlimb; 3, dual hindlimb paralysis; 3.5, partial forelimb paralysis; 4, severe forelimb paralysis; 4.5, immobility; 5, moribund. Mice were sacrificed after displaying paralysis for at least 3 consecutive days. For footpad priming, mice received a total of 100 µg of MOG_35–55_, full-length MOG, or ECD at day 0 (50 µg/hindlimb) and i.p. injections using 300 µl of 1 ng/µl pertussis toxin at days 0 and 2. Mice were sacrificed at day 10 postpriming for downstream analysis.

### Tetramer staining

Mononuclear cells were acquired from processed CNS by collecting the interface of a 70/90 Percoll gradient (Cytiva). Cells were stained for 1 h at room temperature with 4 µg/ml PE- and/or allophycocyanin-conjugated IA^b^:MOG_38–49_ or to IA^b^:CLIP_87–101_ with 1:100 Fc block (BioLegend) in MACS staining buffer (1× Dulbecco’s PBS, 0.5% BSA, 0.5 M EDTA). Samples were washed twice in MACS prior to analysis. Tetramer gates were drawn on a single-sample basis according to IA^b^:CLIP_87–101_ controls such that negative control gates were <1% tetramer-positive. The IA^b^:MOG_38–49_ monomer and tetramer were originally created by the Evavold laboratory (29) and are now provided by the NIH Tetramer Core Facility at Emory University (Atlanta, GA). For tetramer sorting experiments, tetramers were produced in-house using peptide-MHC (pMHC) monomers also provided by the NIH Tetramer Core Facility and following their recommended protocol.

### Enumeration of MOG-specific B cells and T cells

Popliteal and inguinal lymph nodes were pooled and processed after footpad priming. Single-cell suspensions were divided to separately analyze T cells and B cells. For MOG-specific B cell identification, lymphocytes were incubated for 45 min on ice with PE-conjugated (pE/R-phycoerythrin conjugation kit—Lighting-Link, Abcam, ab102918) MOG_1–125_ protein and biotinylated OVA (biotin conjugation kit—Lightning-Link, Abcam, ab201796) to exclude nonspecific binding. After washing twice with 1× PBS, cells were incubated with anti-PE MicroBeads for 15 min according to the manufacturer’s instructions before enrichment of Ag-specific cells using LS columns. Bound and unbound fractions were subsequently stained at 1:300 with Abs against CD19 (SuperBright 780, clone 6D5, Invitrogen), IgD (BV510, clone 11-26, BD Biosciences), IgM (pE-Cy7, clone II/41, Invitrogen), CD38 (Alexa Fluor 700, clone 90, Invitrogen), Fas (Alexa Fluor 488, clone 15A7, Invitrogen), GL-7 (eFluor 450, clone GL7, Invitrogen), and streptavidin allophycocyanin-Cy7 (BD Biosciences). Live, IgD^−^CD19^+^ B cells were quantified from bound and unbound fractions using an Invitrogen Attune NxT flow cytometer. Separate fractions were stained with Zombie NIR (BioLegend) per the manufacturer’s protocol before staining for 30 min on ice at 1:300 with B220 (eFluor 450, clone RA3-6B2, Invitrogen/eBioscience), CD19 (BUV 737, clone ID3, BD Horizon), CD3ε (BUV615, clone 145-2C11, BD OptiBuild), CD4 (BUV 496, clone GK1.5, BD Horizon), CD8α (BV750, clone 53-6.7, BD OptiBuild), CD44 (AF700, clone IM7, BioLegend), CD62L (pE-Cy7, clone MEL-14, BioLegend), PD-1 (BV480, clone 29F.1A12, BD Horizon), CD25 (BV650, clone PC61, BioLegend), and CD127 (BV605, clone A7R34, BioLegend). Live, B220^+^CD19^+^ B cells or TCRβ^+^CD4^+^CD8α^−^ T cells were quantified using a Cytek Aurora. T cells were gated as indicated.

### Cell sorting

CNS mononuclear cells were stained at 1:100 for 30 min on ice with CD4 (allophycocyanin, clone RM4-5, BioLegend), CD8α (BV711, clone 52-6.7, BioLegend), and DAPI (BioLegend). Cells were simultaneously stained at 1:500 with CD90.2 (BV785, clone 30-H12, BioLegend) and CD11b (pE, clone M1/70, BioLegend). CD4^+^ T cells were sorted on a BD FACSAria accordingly (i.e., DAPI^−^CD11b^−^CD90.2^+^CD8α^−^CD4^+^. For tetramer-sorted two-dimensional (2D) analysis, cells were stained with allophycocyanin-conjugated IA^b^:MOG_38–49_ at 4 µg/ml in Fc block for 1 h on ice. At 30 min, the above panel was added as indicated. Cells were washed twice and filtered through a 70-µm cell strainer before analysis on a BD FACSAria. Cells were sorted based on tetramer staining of DAPI^−^CD11b^−^CD90.2^+^CD8^−^CD4^+^IA^b^:MOG_38–49_^+^ T cells. Tetramer-positive T cells were identified using the negative control tetramer IA^b^:CLIP_87–101_.

### 2D MP

A detailed protocol outlining MP is reported elsewhere (42). Briefly, human RBCs were coated with specified densities of IA^b^:MOG_38–49_ or control monomer IA^b^:CLIP_87–101_. Each T cell was randomly selected and probed against a human RBC coated with IA^b^:MOG_38–49_. Binding events were identified upon retraction of the T cell. Each cell was subjected to 50 contact/retraction cycles with the RBC coated at the highest density of monomer. The adhesion frequency (*P*_a_) of individual T cells was recorded. Cells with 0.1 < *P*_a_ < 0.8 were considered Ag reactive and immediately tested to negative control IA^b^:CLIP_87–101_ or IA^b^:NP_311–325_. The CLIP reagent was provided as the negative control from the National Institutes of Health core facility, and the influenza Ag served as a foreign Ag-negative control. Cells with *P*_a_ > 0.8 were tested against a lower density IA^b^:MOG_38–49_ for accurate affinity measurements prior to the negative control. Cells with *P*_a_ < 0.1 were considered nonbinders. The percent Ag-reactive population was determined by (no. cells *P*_a_ > 0.1)/(no. cells tested).

### 2D affinity measurements

The MP independently identifies high- and low-affinity T cells (28). To do so, the adhesion frequency of each tested cell is normalized to the average TCR density of the population and the pMHC density on each RBC. The surface densities of pMHC (ρ_pMHC_) and TCR (ρ_TCR_) were quantified using Abs against I-A/I-E (pE, clone M5/114.15.2, BD Pharmingen) and TCRβ (pE, clone H57-597, BioLegend) based on a linear regression model using Quantibrite PE beads (BD Biosciences). Relative 2D affinity was calculated according to the following equation where *A*_c_ denotes contact area and *K*_a_ denotes the 2D effective affinity: A_c_K_a_(µm^4^) = −ln(*P*_a_)/(ρ_TCR_ × ρ_pMHC_).

### Confocal imaging

Lymph nodes were frozen, sectioned, stained, and imaged as previously described (43). Briefly, the draining lymph node was collected from paralyzed mice and frozen at −20°C in Tissue-Tek OCT compound (Sakura Finetek) until analysis. Sections (10 µm thick) were cut using a Leica CM 1950 (Leica). Slides were fixed using a mixture of acetone and ethanol before being blocked for 1 h with a mix of 5% rat and 5% rabbit serum. Primary staining occurred overnight with the following Abs: CD4 (allophycocyanin, clone RM4-5, Invitrogen) and B220 (FITC, clone RA3-6B2, Invitrogen). The next morning, slides were washed five times in PBS and imaged with an SP8 DIVE (deep in vivo explorer) (Leica).

### Statistical analysis

Flow cytometry data were analyzed using FlowJo v10.10.0 (BD Life Sciences). Cell frequencies and quantification data were imported into and analyzed using GraphPad Prism v10.0.0 for Mac OS X (GraphPad Software). Affinity data were log transformed to increase normality before ANOVA with Tukey posttests (three-sample comparisons) or nonparametric *t* tests (two-sample comparisons) using R statistical programming software (44). To further characterize the data and confirm that pooling samples did not have an effect on the data, a mixed-linear model using lme4 and lmerTest were applied (45, 46). The model consisted of log-transformed affinity data as the response, and a fixed variable of the priming condition with an interaction term of paralysis onset. Any variation due to polling was included as a random effect. Confocal images were processed using ImageJ.

## Results

### Full-length MOG immunization resembled ECD-induced adaptive immune responses

**FIGURE 1.**
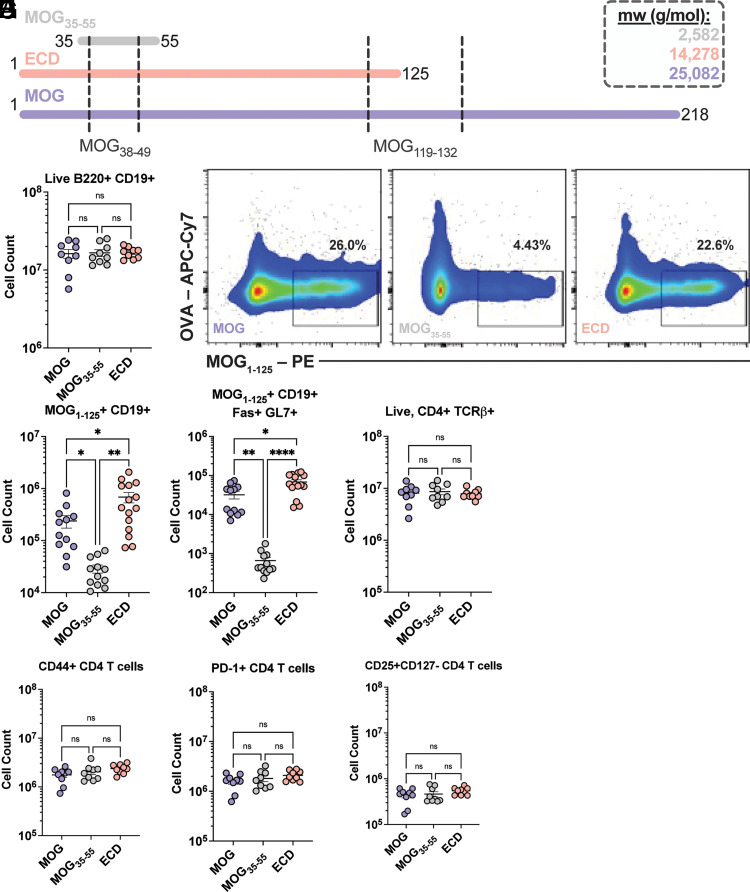
Peripheral B and T cell responses to MOG immunogens. (**A**) Visual depiction comparing sequence lengths of MOG_35–55_ (gray), ECD (pink), and MOG (purple). Encephalitogenic class II IA^b^-restricted epitope regions are indicated by dotted black lines. (**B**) Total CD19^+^ B cells were quantified by flow cytometry at day 10 postimmunization. Each point indicates one mouse (MOG, *n* = 9; MOG_35–55_, *n* = 9, ECD, *n* = 9). (**C**) Representative flow cytometry plots for the identification of MOG-specific B cells after enrichment with PE-labeled MOG_1–125_ protein. OVA is provided as a negative control. (**D**) Quantification of total CD19^+^ MOG B cells specific for MOG_1–125_ by flow cytometry (MOG, *n* = 15; MOG_35–55_, *n* = 15, ECD, *n* = 15). (**E**) Quantification of total Fas^+^GL7^+^ germinal center B cells specific for MOG_1–125_ by flow cytometry (MOG, *n* = 15; MOG_35–55_, *n* = 15, ECD, *n* = 15). (**F**) Quantification of CD4^+^TCRβ^+^ T cells by flow cytometry (MOG, *n* = 9; MOG_35–55_, *n* = 9; ECD, *n* = 9). (**G–I**) Quantification of CD4 T cells expressing activation marker CD44 (G), Ag-experienced marker PD-1 (H), and regulatory T cell markers CD25^+^CD127^–^ (I) (MOG, *n* = 9; MOG_35–55_, *n* = 9; ECD, *n* = 9). For all analyses, lines indicate arithmetic mean ± SEM. *p <* *0.05, **p <* *0.005, ****p <* *0.00005 (by Brown–Forsythe and Welch ANOVA tests). ns, not significant.

Full-length MOG and ECD were recombinantly produced and purified using anti-FLAG affinity columns. Importantly, full-length MOG contains a second encephalitogenic T cell epitope otherwise absent from ECD and MOG_35–55_ (Fig. 1A). To observe the adaptive immune response to each, mice were footpad challenged with 100 µg of full-length MOG, MOG_35–55_, or ECD, and popliteal and inguinal lymph nodes were analyzed at day 10, as traditionally performed for footpad challenges (28–30). The total number of CD19^+^B220^+^ B cells did not change in response to priming by each immunogen (Fig. 1B). An anti-fluorophore magnetic enrichment strategy was therefore employed using fluorescently labeled MOG_1–125_ protein before quantification by flow cytometry to determine the B cell Ag reactivity of each group (Fig. 1C). Staining with fluorescently labeled OVA protein was used as a negative control. Both full-length MOG and ECD expanded more MOG_1–125_-reactive CD19^+^ B cells compared with MOG_35–55_ (Fig. 1D). Germinal center CD19^+^ B cells were further distinguished as Fas^+^GL7^+^ and were elevated after each protein priming (Fig. 1E). Matured B cell responses after ECD immunizations compared with MOG_35–55_ have been published (47, 48), but have never been reported for full-length MOG.

Upon analysis of the T cell response, no difference in total CD4^+^TCRβ^+^ T cells was observed in the draining lymph nodes after footpad priming (Fig. 1F). Likewise, there was no change in the number of CD44^+^ activated or PD-1^+^ Ag-experienced CD4 T cells (Fig. 1G, 1H). The CD25^+^CD127^−^ regulatory T cell counts were also consistent (Fig. 1I). These data confirm that full-length MOG generates a similar magnitude of peripheral adaptive immunity to ECD.

### B cells are pathogenic during EAE induced by full-length MOG

**FIGURE 2.**
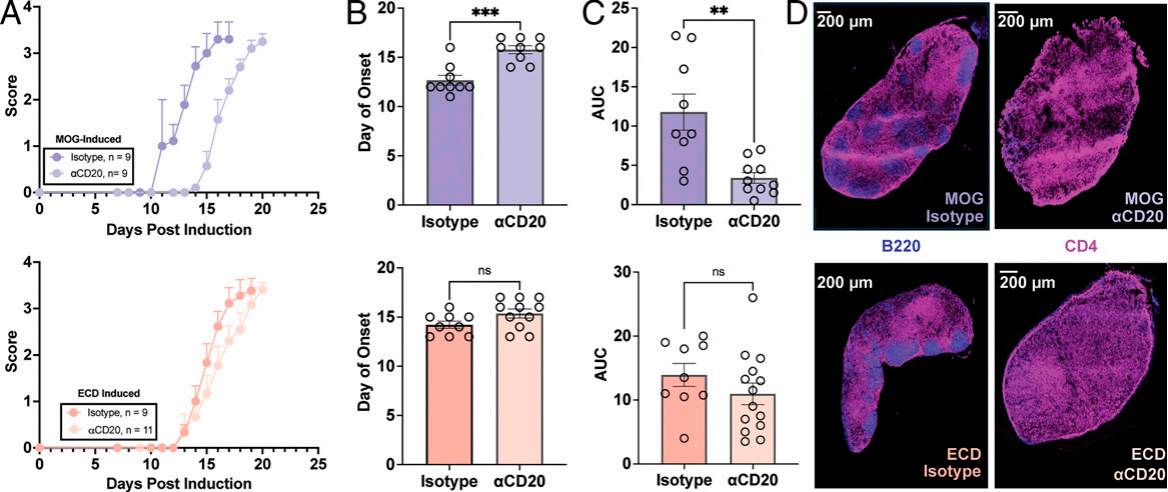
Pathogenic role of B cells during full-length MOG-induced EAE. (**A**) Mice were treated with anti-CD20 or IgG2c isotype control before induction of EAE with MOG (top) or ECD (bottom). Curves shown are from paralyzed mice only. (**B**) Day of paralysis onset was compared between mice treated with anti-CD20 or isotype control before induction of EAE with MOG (top) or ECD (bottom); bars indicate arithmetic mean ± SEM. ***p <* *0.0005 (by unpaired Student *t* test). (**C**) Area under the curve (AUC) was compared between B cell–depleted and control mice after EAE induction by MOG (top) or ECD (bottom); arithmetic mean ± SEM. **p <* *0.005 (by unpaired Student *t* test, Welch correction). (**D**) Representative confocal images of injection site draining lymph nodes that were harvested from mice during peak paralysis after MOG (top) or ECD (bottom) priming. B220^+^ (blue) B cells and CD4^+^ (pink) T cells are indicated. ns, not significant.

EAE was induced with either 100 µg of full-length MOG or ECD after treatment with anti-CD20 or IgG2c isotype control to determine the impact of B cells. Anti-CD20 treatment dramatically delayed paralysis after full-length MOG priming (15.8 ± 1.2 versus 12.7 ± 1.5 d, *p =* 0.002) (Fig. 2A, 2B, top panels) but did not change the onset of ECD-primed mice (15.4 ± 1.5 d versus 14.2 ± 1.1 d, *p =* 0.07) (Fig. 2A, 2B, bottom panels). The delay resulted in a cumulatively milder full-length MOG-induced disease (area under the curve of 3.4 ± 0.7 versus 11.8 ± 2.3, respectively, *p* = 0.0019) (Fig. 2C, top panel) that was not observed for ECD-induced mice (area under the curve of 4.8 ± 1.2 versus 7.4 ± 1.2, respectively, *p* = 0.15) (Fig. 2C, bottom panel). Confocal microscopy images taken from the injection draining lymph nodes of paralyzed mice confirmed that the B cells were depleted even at 4 wk posttreatment (Fig. 2D, blue) despite the continued presence of CD4^+^ T cells (Fig. 2D, pink). Thus, B cells exacerbate EAE after full-length MOG priming, but have limited influence during ECD-primed EAE.

### Full-length MOG-induced EAE reduced the frequency of MOG_38__–__49_-reactive, low-affinity T cells

**FIGURE 3.**
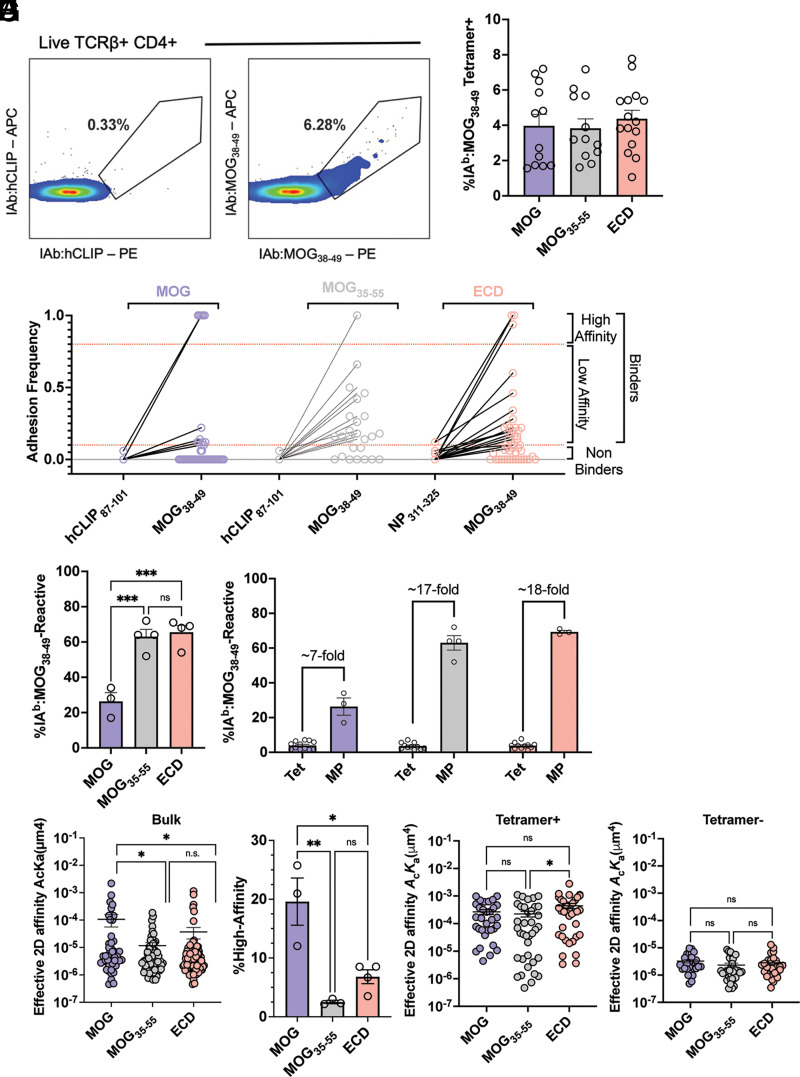
MOG_38__–__49_-reactive T cell response to full-length MOG in the CNS. (**A**) CNS was harvested from paralyzed mice, and mononuclear cells were stained with negative control IA^b^:CLIP_87–101_ tetramer (left flow plot) or IA^b^:MOG_38–49_ tetramer (right flow plot) before analysis by flow cytometry. Representative plots (left) and summary data (right) of IA^b^:MOG_38–49_-reactive T cells identified in the CNS of paralyzed mice during EAE are shown. Each point represents an individual mouse, and bars indicate the arithmetic mean ± SEM. Not significant (by ordinary one-way ANOVA). (**B**) Representative depiction of IA^b^:MOG_38–49_ reactivity determined by MP. Each point represents an individual T cell’s adhesion frequency to indicated monomer, and lines connect the same T cell’s adhesion frequency with each monomer. Each induction strategy is indicated above tested pairs, and general affinity ranges are indicated to the right of the graph. (**C**) MP-determined frequency of IA^b^:MOG_38–49_-reactive CD4^+^ T cells sorted from the pooled CNS of paralyzed mice. Each individual point signifies the frequency detected in an individual experiment from pooled mice. Bars indicate arithmetic mean ± SEM. ***p < 0.0005 (by ordinary one-way ANOVA). (**D**) Fold increase of MOG_38–49_-reactive CD4^+^ T cells determined by MP as compared with tetramer analysis replotted from (A) and (C) to indicate differences in fold increases. (**E**) MP was used to determine TCR:pMHC affinity for CD4^+^ T cells in (B) against IA^b^:MOG_38–49_. *p <* *0.05 (by ordinary one-way ANOVA with Tukey comparison for log-transformed affinity values) (**F**) Percent of tetramer-positive T cells from total MOG_38–49_-reactive T cells, calculated as (average % tetramer-positive)/(% MOG-reactive by MP). Data points represent average fraction tetramer-positive from each experiment ± SEM. *p <* *0.05, **p <* *0.005 (by ordinary one-way ANOVA). (**G** and **H**) CD4 T cells were sorted into groups of IA^b^:MOG_38–49_ tetramer-positive (G) or tetramer-negative (H) before 2D affinity measurements by MP. *p <* *0.05 (by ordinary one-way ANOVA with Tukey comparison for log-transformed affinity values). ns, not significant.

To investigate how multiple epitopes within MOG affect the distribution of encephalitogenic MOG_38–49_-reactive T cells, EAE was induced with 200 µg of MOG_35–55_ or 100 µg of full-length MOG or ECD. An increased amount of peptide was required to break tolerance such that all groups had similar scores of ∼3 upon analysis (Supplemental Fig. 1). The IA^b^:MOG_38–49_ tetramer identified a low frequency (∼4%) of CD4^+^ T cells after each induction (Fig. 3A). This frequency underappreciates the total frequency of MOG_38–49_-reactive T cells (28, 30). Thus, the 2D MP was used to more accurately assess the frequency of MOG_38–49_-reactive T cells inclusive of the low-affinity TCRs. CD4 T cells identified by MP showed Ag specificity by their exclusive binding to IA^b^:MOG_38–49_ and lack thereof to control epitopes IA^b^:CLIP_87–101_ and IA^b^:NP_311–325_ (Fig. 3B). MOG_35–55_ and ECD priming resulted in ∼60 or ∼65% MOG_38–49_-reactive CNS-homing CD4 T cells by MP (Fig. 3C). MP therefore has a remarkable increase in detection compared with tetramer staining (17- and 18-fold for MOG_35–55_ and ECD, respectively) (Fig. 3D). In stark contrast, only ∼30% of responding T cells were MOG_38–49_ reactive after full-length MOG induction (Fig. 3C), which yielded only a 7-fold increase in detection by MP (Fig. 3D). The fold difference between the percentage of Ag-reactive cells identified by tetramer (high-affinity TCRs) and those identified by MP (both high- and low-affinity TCRs) reflects the frequency of low-affinity T cells identified by MP, suggesting that MOG_35–55_ and ECD promote more low-affinity T cells compared with full-length MOG. Indeed, the average 2D TCR affinity for MOG_38–49_ was increased after full-length MOG induction relative to MOG_35–55_ and ECD (Fig. 3E). The affinity measurement also significantly increased statistical variance due to a differential proportion of high- to low-affinity T cells (Fig. 3E). Another means for comparing high- to low-affinity T cells is by considering the percent tetramer-positive out of the total frequency of T cells reactive toward MOG_38–49_. By this analysis, mice induced with full-length MOG had a greater frequency of tetramer-positive T cells in the CNS (19.6 ± 4.0%) compared with those induced with MOG_35–55_ (2.5 ± 0.3%) and ECD (6.8 ± 1.2%) (Fig. 3F). No statistical difference was observed between MOG_35–55_ and ECD (Fig. 3F). Affinity differences are either a consequence of a change in the ratio of high- to low-affinity T cells or an overall change in affinity profile. T cells were therefore FACS sorted into tetramer-enriched and tetramer-negative populations to compare higher- and lower-affinity cells separately. No change was observed between full-length MOG-induced cells and MOG_35–55_- or ECD-primed cells for either high- or low-affinity populations (Fig. 3G, 3H). The tetramer enrichment sort resulted in some low-affinity T cells from the MOG_35–55_-induced group compared with ECD (Fig. 3G) due to a less stringent tetramer gating strategy than previously reported (Supplemental Fig. 2) (28). Ultimately, the data indicate that full-length MOG priming reduced the frequency of low-affinity MOG_38–49_-reactive, encephalitogenic T cells during EAE.

### B cell depletion restored the dominance of encephalitogenic MOG_38__–__49_-reactive T cells

**FIGURE 4.**
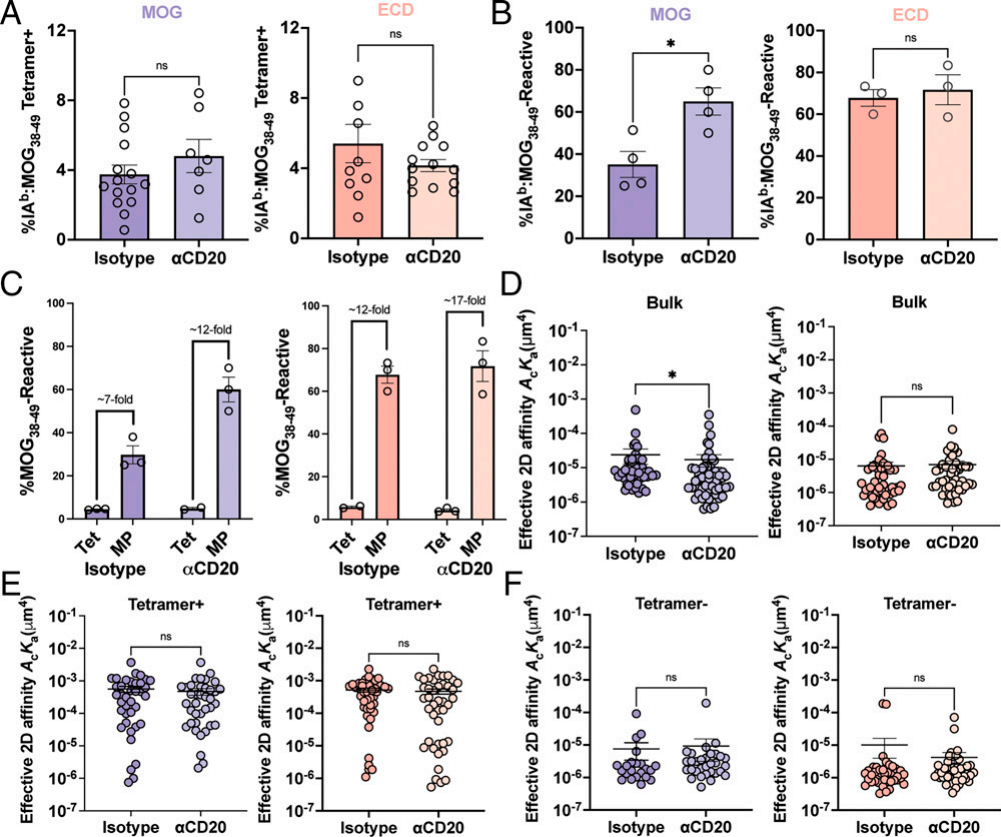
B cell influence over CNS localized T cells reactive to MOG_38__–__49_ during full-length MOG-induced EAE. (**A**) Summary plots of tetramer-positive CD4^+^ T cells in the CNS of paralyzed mice receiving the indicated treatment and inductions. Each point represents an individual mouse, and bars indicate the arithmetic mean ± SEM. Not significant (by ordinary one-way ANOVA). (**B**) MP-determined frequency of IA^b^:MOG_38–49_-reactive CD4^+^ T cells sorted from the pooled CNS of paralyzed mice. Each individual point signifies the frequency detected in an individual experiment from pooled mice. Bars indicate arithmetic mean ± SEM. *p <* *0.05 (by Student *t* test). (**C**) Fold increase of MOG_38–49_-reactive CD4^+^ T cells determined by MP as compared with tetramer analysis replotted from (A) and (B) to emphasize differences in fold increases. (**D**) 2D affinities of CD4 T cells from paralyzed mice treated with anti-CD20 or isotype prior to induction with MOG (left) or ECD (right). *p <* *0.05 (by Student’s *t* test with Tukey comparison for log-transformed affinity values). (**E** and **F**) 2D TCR:pMHC affinity of tetramer-positive (E) or tetramer-negative (F) CD4^+^ T cells sorted from the CNS of paralyzed mice. Each data point indicates the 2D affinity of an individual T cell as determined by the MP. The tested populations include CD4 T cells from anti-CD20– or isotype control–treated, paralyzed mice induced with MOG (left) or ECD (right). Affinity values were log-transformed before a Tukey test. ns, not significant.

To investigate how B cells may have altered the composition of encephalitogenic, MOG_38–49_-reactive CD4 T cells, B cells were depleted prior to induction of EAE with either full-length MOG or ECD to evaluate their impact on MOG_38–49_-reactive CD4 T cells. Treatment with anti-CD20 before priming did not affect the paralysis score recorded during analysis (Supplemental Fig. 1). There was no difference in the percentage of tetramer-positive T cells in B cell–depleted mice after EAE induction with either full-length MOG or ECD (Fig. 4A). However, by MP, B cell depletion markedly increased the frequency of CNS-homing T cells recognizing MOG_38–49_ (from ∼30 to ∼60%) (Fig. 4B, left). In contrast, B cell depletion did not alter the frequency of T cells reactive for MOG_38–49_ from ECD-induced EAE (65 versus 65%) (Fig. 4B, right). Identical to untreated mice in Fig. 3D, only an ∼7-fold increase in MOG_38–49_-reactive CD4 T cells was identified by MP compared with tetramer when mice were pretreated with isotype control prior to full-length MOG-induced EAE (Fig. 4C, left, isotype). In contrast, B cell–depleted mice showed an increase in T cells identified by MP to ∼12-fold (Fig. 4C, left, anti-CD20), which is similar to ECD- and MOG_35–55_-induced mice observed in Fig. 3D. For ECD-induced EAE, both B cell–sufficient and –deficient mice both reported an elevated >12-fold increase in MOG_38–49_-reactive CD4 T cells (Fig. 4C, right), which replicated fold differences observed for MOG_35–55_ and ECD induction in Fig. 3D in addition to B cell–depleted, full-length MOG-induced EAE in Fig. 4C. Anti-CD20 treatment also decreased the net TCR affinity of CD4 T cells for MOG_38–49_ after full-length MOG-induced EAE (Fig. 4D, left), whereas there was no affect in the ECD-induced mice (Fig. 4D, right). In concordance with Fig. 3E, the TCR affinity from isotype-treated, full-length MOG-induced mice was significantly greater than that of the ECD-induced group (Fig. 4D, isotype versus isotype, *p *<* *0.0001). On analysis of the tetramer-enriched and tetramer-negative populations, there was no change in affinity as a result of anti-CD20 treatment before EAE induction with either full-length MOG or ECD (Figs. 4E, 4F). The data collectively demonstrate that full-length MOG-primed EAE reduces the frequency of low-affinity, MOG_38–49_-reactive T cells only when B cells are present.

## Discussion

Induction of IA^b^-restricted B6 mice with the encephalitogenic MOG_35–55_ peptide has been one of the most used EAE models since its inception (10). Much of the focus on this model has been on the CD4 T cell response, which in B6 mice mostly consists of CD4 T cells reactive toward MOG_38–49_ (30, 34). The induction of EAE with myelin peptides as opposed to proteins, however, limits the capacity to study the effects of T cell epitope diversity. Additionally, a regulatory B cell phenotype is observed after MOG_35–55_ peptide-induced EAE (49–51), but protein-primed EAE expands pathogenic B cells (25, 47, 48, 52–59). Therefore, protein-primed EAE could serve as a model to decode the recent success of therapies to target B cells during MS.

The aim of this study was to assess the effects of full-length MOG on T cell epitope reactivity in the CNS. Each MOG immunogen encompasses MOG_38–49_, rendering it as a point of comparison between full-length MOG, ECD, and MOG_35-55_. There is a dramatic reduction in the frequency of MOG_38–49_-reactive CD4 T cells in the CNS during EAE induced by full-length MOG relative to MOG_35–55_ and ECD (Fig. 3). Full-length MOG differs from both reagents in that its additional sequence (aa 126–218) provides a second encephalitogenic epitope (39, 40). The presence of this additional epitope would explain the decrease in encephalitogenic MOG_38–49_-reactive T cells found in the CNS. While monitoring transmembrane-reactive T cells and their TCR kinetics is of interest, the exceedingly hydrophobic nature of MOG_119–132_ has prevented the generation of stable pMHC monomers for tetramer or single-cell MP analysis at this time.

How multiple self-epitopes affect autoimmune T cell expansion kinetics is relatively unexplored. EAE induction with multiepitope full-length MOG reduces the overall frequency of T cells reactive for MOG_38–49_ (Fig. 3), suggesting competition between the epitopes. In B6 mice, T cells that recognize MOG_38–49_ with low affinity dominate the naive and activated repertoires, and these populations expand with similar kinetics in a single-epitope setting (31). The expansion kinetics of high- and low-affinity T cells are equivalent between peptide and ECD as evidenced by their identical proportion of high- to low-affinity T cells (Fig. 3). However, a reduced frequency of low-affinity T cells was detected after full-length MOG priming (Fig. 3), which returned to normal levels after targeting B cells (Fig. 4). Each of these observed changes in frequency occurs independent of net affinity changes (Figs. 3, 4). Taken together, these results demonstrate that the frequency of low-affinity, MOG_38–49_-reactive T cells in the CNS are limited by the presence of an encephalitogenic T cell epitope uniquely found in full-length MOG.

Many murine EAE studies implicate B cell pathogenicity by modulating the pathogenic CD4 T cell response (25, 53–56). In this study, B cell depletion restores the frequency of CNS-derived MOG_38–49_-reactive T cells during full-length MOG-induced EAE (Fig. 4) to levels observed after single-epitope immunizations (Figs. 3, 4). This effect could be due to a loss of Ab-mediated CD4 T cell entry into the CNS (53). However, ECD priming also induces MOG-specific B cells and their depletion does not change the detection of MOG_38–49_-reactive T cells (Fig. 4). This suggests Ag presentation as a possible mechanism as indicated by several other studies (25, 47, 52, 56–58). Regardless, our data demonstrate that B cells can shift the MOG_38–49_-reactive T cell response. Such B cell–mediated control of CNS-derived epitope diversity is also implicated during MS (5, 19, 20), EAE (21–25), and thymic selection (60).

In conclusion, there was a reduced frequency as well as a reduction in lower-affinity, tetramer-negative T cells that recognize MOG_38–49_ after immunization with full-length MOG protein. Use of anti-CD20 to remove B cells restored T cell reactivity to MOG_38–49_ to an identical frequency observed during MOG_35–55_ and ECD immunizations. Both MOG_35–55_ and ECD contain a single encephalitogenic epitope, but only the full-length MOG sequence encodes an additional encephalitogenic T cell epitope (39, 40). The straightforward explanation is that the loss of lower-affinity T cells to MOG_38–49_ was caused by the presence of an additional epitope found in full-length MOG as compared with ECD. Anti-CD20 could instead remove a non–B cell population expressing CD20 or affect T cells by an Ab-dependent process (53, 61). The precise mechanism remains to be defined. Ultimately, this study shows that anti-CD20 treatment affected encephalitogenic T cells found in the CNS during demyelinating disease.

## Supplementary Material

Supplemental Material (PDF)
