# Supplementary material for: B Cells Influence Encephalitogenic T Cell Frequency to Myelin Oligodendrocyte Glycoprotein (MOG)38–49 during Full-length MOG Protein–Induced Demyelinating Disease
Source: Immunohorizons. 2024 Sep 27;8(9):729–39. doi: 10.4049/immunohorizons.2400069 (PMC11447661; doi:10.4049/immunohorizons.2400069)
Supplement: Supplemental Material (PDF) [file IH_2400069_Supplemental_3.pdf]

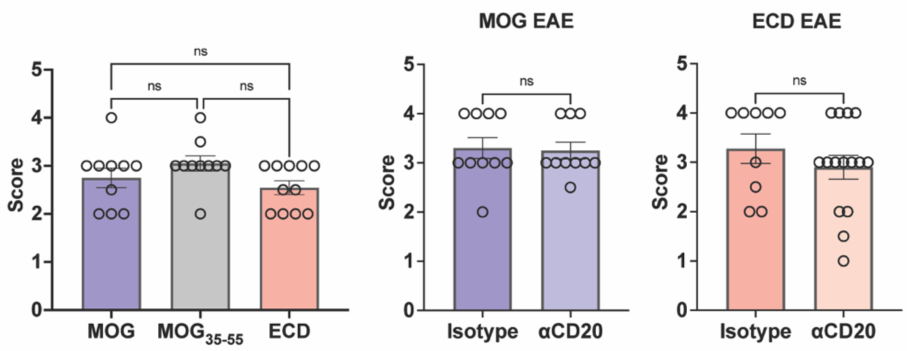

Supplemental Figure 1. Mouse paralysis scores at time of analysis of T cell Ag reactivity.

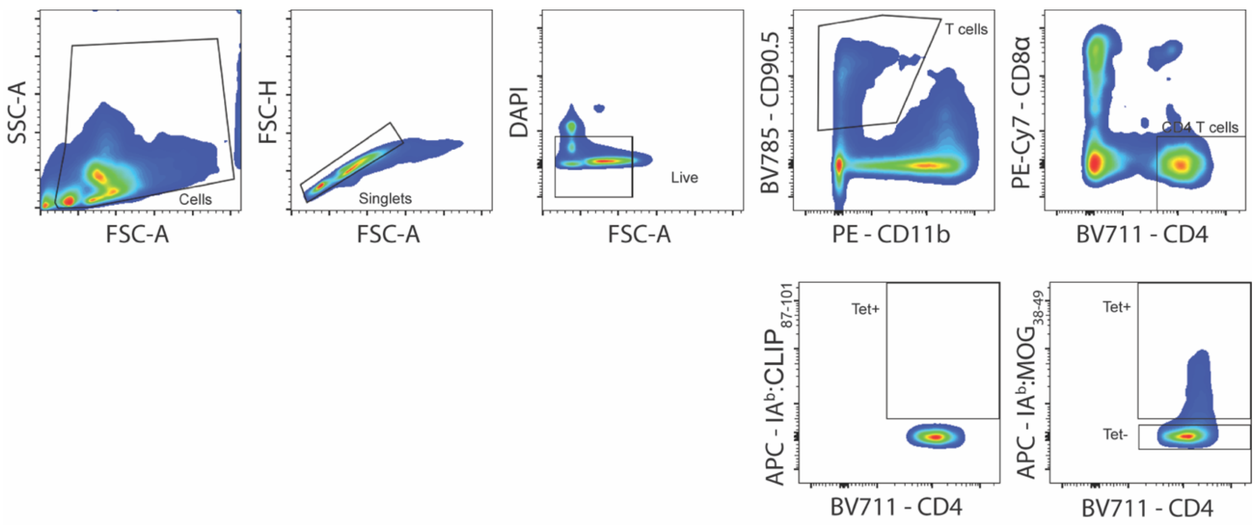

Supplemental Figure 2. IA^b:MOG$_{38\text{-}49}$ tetramer gating strategy for affinity analysis.
